# Emotional exhaustion predicts poor sleep quality among orthopedic nurses: a cross-sectional study

**DOI:** 10.3389/fpubh.2025.1711331

**Published:** 2026-01-22

**Authors:** Jiawen Tan, Lan Lei, Fulong Liu

**Affiliations:** 1 Department of Rehabilitation, Sichuan Provincial Orthopedic Hospital, Chengdu, China; 2 Human Movement Science, Sichuan Sports College, Chengdu, China

**Keywords:** burnout, emotional, job burnout, orthopedic nurse, sleep quality

## Abstract

**Objective:**

The purpose of this study was to explore the effect of orthopedic nurse burnout on sleep quality.

**Methods:**

The Chinese versions of the Maslach Burnout Inventory–General Survey and the Pittsburgh Sleep Quality Index (PSQI) were administered to 184 orthopedic nurses in five Grade-A tertiary hospitals in Chengdu. Multiple linear regression was used for data analysis.

**Results:**

The total PSQI score of orthopedic nurses was 6.10 ± 2.87, and the detection rate of sleep disorders was 58.7%; the total job burnout score was 43.14 ± 9.76, and emotional exhaustion, depersonalization, and reduced personal accomplishment were all moderately positively correlated with PSQI (*r* = 0.228–0.376); regression showed that the three dimensions jointly explained 41.7% of the variance in sleep quality, with emotional exhaustion (β = 0.301) having the largest effect; being married, having more than 5 years of work experience, and low income also significantly positively affected sleep quality.

**Conclusion:**

Job burnout significantly affects sleep quality among orthopedic nurses, with emotional exhaustion having the greatest impact; those who are married, have long tenure, and have low income are at higher risk. Reducing burnout and strengthening support and incentives are key to improving sleep quality among orthopedic nurses.

## Introduction

Sleep, as one of the basic physiological needs of humans, is directly related to physical and mental health, work efficiency, and quality of life (1, 2). For medical staff, good sleep is crucial for efficient performance, accurate judgment, and high-quality nursing services (3, 4). However, due to the particularity of nursing profession (such as shift work, high intensity work, high psychological stress, etc.), nurses generally suffer from sleep disorders (5), and a meta-analysis found that the proportion of sleep disorders among 3,499 intensive care nurses was as high as 75.1% (6). Orthopedic patients often have complex conditions, long courses of disease, long postoperative rehabilitation cycles, and high risk of complications (7), which requires nurses not only to have solid professional knowledge and skills but also to invest a lot of physical strength and energy in patient care, such as assisting patients in turning over, traction care, rehabilitation training guidance, etc. (8, 9). In addition, there are many emergency trauma patients in orthopedics, and the condition changes rapidly, so nurses need to maintain high vigilance at all times, and their spirit is in a state of tension for a long time (10). The superposition of these factors makes orthopedic nurses a high-risk group for job burnout and sleep problems.

Job burnout refers to a comprehensive psychological response of physical and mental fatigue, emotional exhaustion, depersonalization, and reduced personal accomplishment that occurs under long-term work pressure (11, 12). Job burnout not only leads to a decrease in nurses' work enthusiasm and quality of care but also causes serious damage to their physical and mental health, increases turnover intention, and thus affects the stability of the nursing team and the continuity of medical services (13, 14). As an important indicator of physical and mental health, sleep quality is closely related to job burnout (15). On the one hand, job burnout can trigger negative emotions such as anxiety and depression, interfere with normal sleep rhythm, and aggravate problems such as difficulty falling asleep, sleep maintenance disorders, and early awakening (16); on the other hand, the decline in sleep quality will further aggravate individual fatigue and emotional exhaustion, reduce their ability to cope with work pressure, and form a vicious circle (17).

In recent years, scholars have paid widespread attention to the job burnout and sleep quality of nurse groups (6, 18–22). Studies have mostly focused on the sleep status and influencing factors of nurses in different departments, such as intensive care units (ICU), emergency departments, and other high-pressure departments, and found that nurses in these departments have a significantly higher incidence of sleep disorders than ordinary department nurses due to the tense work environment and critical patient conditions (6, 18). At the same time, research has focused on exploring the correlation between nurses' job burnout and sleep quality, as well as the impact of demographic variables (such as age, years of service, marital status, etc.) on sleep quality (19, 20). Older nurses, those with longer years of service, and married nurses often face more dual pressures from family and work, and their sleep quality is relatively poor (21). Nurses with higher monthly incomes may have better living conditions and sleep environments due to better economic conditions, and their sleep quality is relatively better (22).

Existing studies have examined various sleep problems among nurses. However, there are the following shortcomings in current research. Studies have focused on nurses in departments such as ICU, psychiatry, and emergency (6, 18), but there has been insufficient attention to orthopedic nurses, specifically. Additionally, studies have mostly examined overall job burnout as associated with overall sleep quality (19, 20). There has been a lack of in-depth analysis of the relationship between each dimension of job burnout and each dimension of sleep quality.

This study aims to reveal the relationship between job burnout and sleep quality among orthopedic nurses, and to provide a scientific basis for clinical nursing managers to formulate precise psychological intervention measures, improve nurses' sleep quality, and reduce job burnout levels. The study seeks to examine whether each dimension of job burnout among orthopedic nurses is correlated with total sleep quality scores.

## Participants and methods

### Participants

This study was approved by the Ethics Committee of Sichuan Sports College (SSC202509). A cross-sectional survey design was adopted, and the study was conducted in the orthopedic departments of five tertiary grade-A hospitals (the highest level of hospitals in China's hospital accreditation system) in Chengdu from August to September 2025. Inclusion criteria: holding a nurse practice certificate of the People's Republic of China; having worked continuously in orthopedic clinical nursing for 1 year or more; being 18 years of age or older; the study complies with the Declaration of Helsinki, and informed consent was signed.

Exclusion criteria: currently pregnant, given birth (or adopted an infant) within the past 3 months, currently breastfeeding, or being in a post-miscarriage recovery period; currently taking sedative-hypnotic drugs or receiving treatment for sleep disorders; having a history of severe mental illness; having experienced any major life events within the past month.

According to Kendall's sample size estimation principle, the sample size required for multivariate regression analysis should be 5–10 times the number of independent variables. This study included a total of 10 independent variables (three for job burnout, one for sleep quality, and six for demographics), calculated at 10 times and considering a 10% invalid questionnaire, the sample size was determined to be 100 cases. Finally, questionnaires were distributed through convenience sampling. Specifically, via the online Wenjuanxing platform, the head nurses of orthopedic departments in each cooperating hospital forwarded the questionnaire link to the WeChat group of department nurses, and questionnaires were collected from orthopedic nurses in the group who met the inclusion criteria, all in the form of anonymous electronic questionnaires without offline distribution.

A total of 192 questionnaires were distributed in this study. Invalid questionnaires included eight questionnaires with incomplete filling and obvious logical contradictions in answers, so a total of 184 valid questionnaires were finally included in the statistical analysis. All included subjects were registered orthopedic nurses, including 156 females (84.8%) and 28 males (15.2%); 56 were aged ≤ 25 years, 98 were aged 26–35 years, and 30 were aged > 35 years; 112 were married (60.9%), and 72 were unmarried (39.1%); 80 had worked for ≤ 5 years, and 104 had worked for > 5 years; in terms of professional titles, there were 122 ordinary nurses and 62 head nurses or above; 68 had an average monthly income of ≤ 10,000 yuan, 90 had an income of 10,001–15,000 yuan, and 26 had an income of >15,000 yuan. The grouping by sex, age, education, marital status, years of service, professional title, and average monthly income is shown in Table 1.

**Table 1:** Overall scores of job burnout and sleep quality among orthopedic nurses (*n* = 184).

**Variable**	**Score range**	**Mean ±standard deviation**
Emotional exhaustion	0–30	18.65 ± 5.52
Depersonalization	0–24	8.42 ± 3.31
Reduced personal accomplishment	0–36	16.07 ± 4.89
Total burnout score	0–90	43.14 ± 9.76
PSQI score	0–21	6.10 ± 2.87
Sleep disturbance [*n* (%)]	—	108 (58.7)

### Methods

#### General information survey

The content includes sex, age, education, marital status, years of service, professional title, average monthly income, employment form, and night shift frequency, etc. The questionnaire was evaluated by five nursing experts, and the content validity index was 0.92, indicating good content validity.

#### Maslach burnout inventory-general survey (MBI-GS) Chinese version test

The MBI-GS (23) is a widely used general burnout measurement scale. With the authorization of the scale developer Professor Leiter, Chinese scholar Li (24) revised and introduced it: first, it was translated from English to Chinese by experts, and the expressions were adjusted based on participant interviews, and then it was back-translated and optimized based on Professor Leiter's opinions, and a 16-item Chinese version was determined; later, one item with high cross-loading in the “cynicism” dimension was deleted, and a final 15-item version was formed, which has the same structure as the original version and has good structural validity. The 15-item Chinese version includes three dimensions with clear sub-items and definitions: Emotional exhaustion (five items, e.g., Work makes me emotionally exhausted)—defined as a psychological state of emotional resource depletion, mental fatigue, and loss of work enthusiasm caused by long-term work pressure, which is the core dimension of job burnout; Cynicism (four items, e.g., I have become indifferent to patients needs)—assessing coldness and alienation toward work objects; Reduced personal accomplishment (6 items, e.g., I rarely feel a sense of accomplishment at work)—evaluating the decline in work competence and sense of value. The scale uses a 0–6 point seven-level scoring system (0 = never, 6 = very frequently), with the reduced personal accomplishment dimension being reverse-scored and the other two dimensions being positively scored; higher scores indicate greater burnout. In Li's (24) study, the internal consistency coefficients of the three dimensions were 0.88, 0.83, and 0.82, respectively. In this study, the Cronbach's α coefficients of each dimension were 0.882, 0.834, and 0.861, respectively, and that of the total scale was 0.876, indicating good internal consistency.

#### Pittsburgh sleep quality index (PSQI) test

It includes 18 items and assesses seven dimensions with clear definitions and evaluation criteria: Subjective sleep quality (one item): Overall self-evaluation of sleep quality; Sleep onset latency (two items): Time from lying down to falling asleep (>30 min is considered abnormal); Sleep duration (one item): Actual nightly sleep time (< 6 h is considered abnormal); Sleep efficiency (two items): Percentage of actual sleep time relative to time in bed (< 85% is considered abnormal); Sleep disturbances (nine items): Frequency of nighttime awakenings, snoring, apnea, and other sleep-interfering factors; Use of hypnotic drugs (one item): Frequency of hypnotic drug use in the past month; Daytime dysfunction (two items): Degree of daytime fatigue, inattention, and other problems caused by insufficient sleep. Each dimension is scored 0–3 points, with a total score ranging from 0 to 21; a score >5 indicates the presence of sleep disorders (25, 26). In this study, the Cronbach's α coefficient of the PSQI scale was 0.908, and the reliability coefficients of each dimension ranged from 0.71 to 0.85, showing good reliability.

#### Questionnaire distribution form and quality control

Data collection was conducted through the Wenjuanxing platform, a professional online survey tool widely used in Chinese academic research with verified reliability (27). It has passed ISO 27001 information security certification, supports encrypted data storage, and has functions such as logical jump setting and duplicate submission prevention, which can effectively ensure data quality and participant privacy. Before the study, contact was made with the nursing departments of each hospital to obtain consent, and the department head nurses forwarded the questionnaire link to the WeChat group of orthopedic nurses. An informed consent statement was set on the first page of the questionnaire, and participants could enter the filling interface only after checking “agree.” Quality control questions (such as What is the capital of China?) were set in the questionnaire, and those who answered incorrectly were automatically excluded. To achieve anonymous submission and unique response, the Wenjuanxing platform was configured with the following technical settings: Each WeChat account and IP address was restricted to one submission only to prevent duplicate filling; No personally identifiable information (e.g., name, employee ID) was included in the questionnaire to ensure anonymity; The system automatically recorded submission devices and IP addresses for cross-verification. Additionally, the filling time was required to be more than 1 min, and those who took less than this time were considered invalid questionnaires. These measures ensured both participant privacy and data uniqueness.

In terms of quality control, the study strictly followed standardized procedures. All research tools used were widely applied scales with good reliability and validity, ensuring the scientificity and reliability of the measurement results. During the data collection process, logical jumps and required items were set through the electronic questionnaire system to reduce missing and incorrect fillings. In the data entry stage, a unified Excel template was used for coding, and two researchers independently entered and checked the data to ensure accuracy.

### Statistical methods

SPSS 26.0 software was used for data analysis. The Shapiro-Wilk test was used for normality testing, and measurement data were expressed as mean ± standard deviation. Group comparisons were performed using *t*-tests or analysis of variance, correlations between variables were analyzed using Pearson correlation analysis, and multivariate analysis was performed using stepwise multiple linear regression analysis, with a significance level of α = 0.05.

## Results

### Overall levels of job burnout and sleep quality among orthopedic nurses

The Shapiro-Wilk test showed that the data were normally distributed. The total job burnout score of the 184 orthopedic nurses was 43.14 ± 9.76, with the emotional exhaustion dimension scoring 18.65 ± 5.52, the depersonalization dimension scoring 8.42 ± 3.31, and the reduced personal accomplishment dimension scoring 16.07 ± 4.89. The total PSQI score was 6.10 ± 2.87 (minimum score 1, maximum score 15). Using a PSQI score > 5 as the criterion for sleep disorders, the prevalence of sleep disorders in this group of nurses was 58.7% (108/184; Table 1). Referring to the job burnout judgment criteria of the MBI-GS scale, the detection rate of high burnout in the emotional exhaustion dimension among orthopedic nurses in this study was 68.5% (126/184), the detection rate of high burnout in the depersonalization dimension was 52.2% (96/184), and the detection rate of high burnout in the reduced personal accomplishment dimension was 47.8% (88/184). In terms of overall burnout, the proportion of nurses with high burnout in two or more dimensions was 51.1% (94/184).

### Analysis of differences in sleep quality among nurses with different characteristics

Univariate analysis showed that age, marital status, years of service, professional title, and average monthly income had statistically significant differences in PSQI scores (*P* < 0.05). Among them, the PSQI score of the > 35 years group (6.95 ± 3.05) was higher than that of the 26–35 years group (6.31 ± 2.90) and the ≤ 25 years group (5.42 ± 2.71; *F* = 3.214, *P* = 0.041); the score of married nurses (6.59 ± 2.94) was higher than that of unmarried nurses (5.38 ± 2.65; *t* = 2.837, *P* = 0.028); the score of the > 5 years of service group (6.78 ± 2.96) was higher than that of the ≤ 5 years group (5.21 ± 2.58; *t* = 2.456, *P* = 0.032). In terms of professional title, the PSQI score of head nurses and above (6.91 ± 3.10) was higher than that of ordinary nurses (5.89 ± 2.75; *t* = 2.103, *P* = 0.045). The score of the average monthly income ≤ 10,000 yuan group was the highest (6.89 ± 3.10), and that of the > 15,000 yuan group was the lowest (5.12 ± 2.53; *F* = 3.557, *P* = 0.024). There were no statistically significant differences in PSQI scores between sexes and education levels (*P* > 0.05; Table 2).

**Table 2:** Univariate comparison of PSQI scores by demographic characteristics (*n* = 184).

**Characteristic**	**Group**	***n***	**PSQI score**	**Statistic**	***P*-value**
Sex	Male	28	5.79 ± 2.93	*t* = 0.512	0.610
	Female	156	6.15 ± 2.86		
Age (years)	≤ 25	56	5.42 ± 2.71	*F* = 3.214	0.041
	26–35	98	6.31 ± 2.90		
	>35	30	6.95 ± 3.05		
Marital status	Unmarried	72	5.38 ± 2.65	*t* = 2.837	0.028
	Married	112	6.59 ± 2.94		
Years of service	≤ 5	80	5.21 ± 2.58	*t* = 2.456	0.032
	>5	104	6.78 ± 2.96		
Professional	Title staff nurse	122	5.89 ± 2.75	*t* = 2.103	0.045
	Charge nurse and above	62	6.91 ± 3.10		
Monthly income	≤ 10,000	68	6.89 ± 3.10	*F* = 3.557	0.024
	10,001–15,000	90	6.05 ± 2.80		
	>15,000 (ref)	26	5.12 ± 2.53		

Coding: “married = 1,” “unmarried = 0”; “years of service >5 = 1,” “ ≤ 5 = 0.”

### Correlation between job burnout and sleep quality

Pearson correlation analysis showed that emotional exhaustion, depersonalization, reduced personal accomplishment, and the total job burnout score were all significantly positively correlated with the total PSQI score (*r* = 0.358, 0.376, 0.228, 0.392, respectively, *P* < 0.01). For the PSQI dimensions, emotional exhaustion had the strongest correlations with sleep onset latency (*r* = 0.342), sleep efficiency (*r* = 0.318), and daytime dysfunction (*r* = 0.301); depersonalization was closely related to subjective sleep quality (*r* = 0.336) and sleep disturbances (*r* = 0.319); reduced personal accomplishment was moderately correlated with sleep duration (*r* = 0.267) and use of hypnotic drugs (*r* = 0.254). This suggests that the higher the level of job burnout among orthopedic nurses, the worse the performance in each dimension of sleep (Table 3).

**Table 3:** Pearson correlations between burnout dimensions and PSQI global and component scores.

**Dimension**	**Emotional exhaustion**	**Depersonalization**	**Reduced personal accomplishment**	**Total burnout score**
PSQI global score	0.358^**^	0.376^**^	0.228^*^	0.392^**^
Subjective sleep quality	0.289^**^	0.336^**^	0.191^*^	0.313^**^
Sleep latency	0.342^**^	0.318^**^	0.205^*^	0.334^**^
Sleep duration	0.267^**^	0.239^*^	0.267^**^	0.283^**^
Sleep efficiency	0.318^**^	0.295^**^	0.213^*^	0.310^**^
Night-time disturbances	0.301^**^	0.319^**^	0.189^*^	0.308^**^
Hypnotic drug use	0.254^*^	0.231^*^	0.254^*^	0.267^**^
Daytime dysfunction	0.301^**^	0.277^**^	0.176	0.289^**^

^*^P < 0.05, ^**^P < 0.01.

### Multivariate linear regression analysis of factors influencing sleep quality

With the total PSQI score as the dependent variable, variables with *P* < 0.05 in univariate analysis (age, marital status, years of service, professional title, monthly income) and the three dimensions of job burnout were included as independent variables in the stepwise multiple linear regression model. Collinearity diagnostics showed that the variance inflation factors of all variables were < 5, and the tolerance was >0.2, indicating no obvious collinearity. The final model explained 41.7% of the total variance in sleep quality (adjusted *R*^2^ = 0.417, *F* = 9.634, *P* < 0.001).

Regression coefficients showed that emotional exhaustion (β = 0.301, *P* < 0.001), depersonalization (β = 0.275, *P* < 0.001), and reduced personal accomplishment (β = 0.165, *P* = 0.022) had significant positive effects on PSQI scores. The above results indicate that each dimension of job burnout among orthopedic nurses is significantly correlated with worse sleep quality, as measured by higher scores on the PSQI. Among demographic variables, married orthopedic nurses and those with more than 5 years of service had worse sleep quality, while nurses with higher monthly incomes had relatively better sleep quality. Specifically, nurses with monthly income ≤ 10,000 yuan had significantly worse sleep quality than those with monthly income >15,000 yuan (β = −0.141, *P* = 0.034). Being married (β = 0.244, *P* = 0.029) and having more than 5 years of service (β = 0.108, *P* = 0.045) were risk factors for decreased sleep quality, while higher income was a protective factor for sleep quality (Table 4).

**Table 4:** Multiple linear regression analysis of factors influencing sleep quality among orthopedic nurses (*n* = 184).

**Predictor**	***B***	**SE**	**β**	***t***	***P*-value**	**VIF**
Constant	5.923	1.987	—	5.341	< 0.001	—
Emotional exhaustion	1.698	0.512	0.301	3.987	< 0.001	1.89
Depersonalization	1.625	0.453	0.275	3.812	< 0.001	1.76
Reduced personal accomplishment	1.089	0.521	0.165	2.634	0.022	1.93
Marital status (married = 1)	1.512	0.498	0.244	2.876	0.029	1.55
Years of service (>5 = 1)	0.776	0.198	0.108	1.692	0.045	1.62
Monthly income (ref: >15 000)	—	—	—	—	—	—
≤ 10,000	0.823	0.432	0.141	2.387	0.034	1.48
10,001–15,000	0.411	0.398	0.079	1.032	0.305	1.51

Model parameters: F = 9.634, P < 0.001; Adjusted R^2^ = 0.417.

## Discussion

This study surveyed the relationship between job burnout and sleep quality among 184 orthopedic nurses from five tertiary grade-A hospitals in Chengdu. The results showed that the total Pittsburgh Sleep Quality Index score of orthopedic nurses was 6.10 ± 2.87, with 58.7% having sleep disorders; the total job burnout score (43.14 ± 9.76) was at a moderate to high level, and the three dimensions of emotional exhaustion, depersonalization, and reduced personal accomplishment were all significantly positively correlated with the total PSQI score, and could independently explain more than 41% of the variance in sleep quality. The above findings validate the hypotheses proposed in this study, that is, all dimensions of job burnout are positively associated with sleep disorders, and are important influencing factors of sleep quality, with effect sizes higher than those reported for nurses in other departments in the past [the correlation effect value between burnout and sleep disorders among 1,127 general ward nurses was 0.390 (28), and that among 1,044 psychiatric nurses was 0.265 (29)]. It can be seen that in the high-load, high-responsibility, and frequent night-shift occupational context of orthopedics, burnout emotions constitute a risk factor for sleep health, and clinical managers need to give targeted interventions.

From the overall level, the PSQI score of orthopedic nurses (mean 6.1) was significantly higher than the Chinese adult norm (376,824 subjects, mean 4.32) (30), but lower than the meta-analysis result for Chinese nurses (mean 7.13) (31), and the prevalence of sleep disorders (58.7%) was consistent with the 55%−65% range for nurses in the meta-analysis (31), but lower than the 75% level in ultra-high-pressure departments such as intensive care units (6), indicating that although orthopedic nursing is not an extremely high-pressure environment, its sleep damage cannot be ignored. Among the burnout dimensions, emotional exhaustion had the highest score, reflecting that the characteristics of frequent night shifts, trauma emergencies, and rehabilitation make physical fatigue and emotional resource depletion occur simultaneously. Depersonalization and reduced personal accomplishment further affect sleep quality.

Multiple linear regression showed that after controlling for confounding factors such as age, marital status, years of service, and income, emotional exhaustion, depersonalization, and reduced personal accomplishment still significantly positively affected the total PSQI score, indicating that job burnout has a negative impact on sleep. Among them, emotional exhaustion had the largest β value (0.301). When individual emotional resources are continuously consumed without replenishment, nighttime cortisol levels decline delayed, exacerbating difficulty falling asleep and sleep fragmentation (32, 33). Depersonalization was correlated with the use of hypnotic drugs (*r* = 0.254), suggesting that alienation and indifference may prompt nurses to adopt self-help sedative behaviors, which in turn disrupt biological rhythms and further reduce sleep efficiency.

In terms of demographic variables, being married, >5 years of service, and low income all significantly increased PSQI scores, and the effects were independent of the three burnout dimensions. This study believes that married nurses face dual role conflicts between family and work, and in the orthopedic context of “sudden trauma and delayed surgery,” it is more difficult to ensure regular work and rest. Participants with long tenure have accumulated musculoskeletal injuries from long-term physical labor, resulting in more frequent nighttime pain awakenings. Low-income participants experience pre-sleep anxiety due to financial stress, reducing sleep depth. This suggests that intervention programs need to focus not only on psychological burnout but also on family support and salary incentives to comprehensively improve the sleep health of orthopedic nurses.

The results of this study show that even if orthopedic nurses are not in ultra-high-pressure departments such as ICU, the negative effect of job burnout on sleep quality is still as high as 41.7%, with emotional exhaustion being the most prominent. The internal causes can be explained from the following three aspects: First, the characteristics of orthopedic work determine the coexistence of “continuous standby” and “long-term physical exertion,” emergency trauma surgeries are often delayed until late at night, and nurses enter a high-stress recovery stage after work, shortening deep sleep duration (34); Second, the orthopedic rehabilitation cycle is protracted; nurses perform high-intensity turning, traction, and external-fixation care every single day, so physical fatigue and emotional labor accumulate together (10), and intrusive work-related memories surface at night, further lengthening sleep-onset latency. Third, the departments we investigated generally operate a “high-seniority mentoring and low-seniority shift” model: nurses with long tenure must shoulder teaching and quality-control duties in addition to their clinical workload, resulting in marked role overload, yet their performance bonuses are tied to trauma caseload rather than teaching volume. This effort–reward imbalance constitutes a structural source of steadily rising emotional exhaustion and thus keeps their risk of sleep disorders persistently high.

### Clinical implications and intervention strategies

The findings suggest that multi-level comprehensive interventions should target burnout-related sleep problems among orthopedic nurses. First, at the individual level, mindfulness-based stress reduction (MBSR) can be promoted; systematic reviews demonstrate that MBSR significantly reduces emotional exhaustion and improves sleep quality in nurses (35, 36). Second, at the organizational level, scheduling systems should be optimized by implementing a fast-rotation recovery model to ensure at least 48-h recovery periods after consecutive night shifts, limiting night shifts to ≤ 4 per month to reduce circadian disruption. Third, peer support groups should be established, particularly for married and senior nurses facing work-family conflicts, to provide psychological counseling and resource coordination. Finally, management should re-examine performance incentive mechanisms by incorporating non-direct care workloads (e.g., teaching, quality control) into performance evaluations to alleviate effort-reward imbalance among senior nurses. These strategies require validation through cluster randomized controlled trials to assess feasibility and cost-effectiveness in the Chinese orthopedic nursing context. Finally, management should re-examine performance incentive mechanisms by incorporating non-direct care workloads (e.g., teaching, quality control) into performance evaluations to alleviate effort-reward imbalance among senior nurses. Regarding the feasibility of implementing scheduling strategies (e.g., 48-h recovery period after night shifts, ≤ 4 night shifts per month), the 5 Grade-A tertiary hospitals involved in this study have sufficient orthopedic nursing staffing (nurse-to-bed ratio of 1:0.4–0.6, in line with national accreditation standards), providing a personnel basis for flexible scheduling. Moreover, phased implementation (e.g., piloting among high-risk nurses first) can reduce operational pressure. These strategies require validation through cluster randomized controlled trials to assess feasibility and cost-effectiveness in the Chinese orthopedic nursing context.

### Limitations and future research

This study has the following limitations: First, the cross-sectional design cannot determine causal sequence, and future randomized controlled intervention experiments could be added to further verify the causal relationship between burnout and sleep. Second, the PSQI is a self-report tool and may be affected by social desirability; future studies could combine objective assessments with actigraphy or melatonin secretion indicators. Third, the sample was limited to orthopedic departments in five tertiary Grade-A hospitals in Chengdu, restricting the geographical and departmental generalizability of our findings. The healthcare resource allocation, nursing management culture, and night-shift scheduling systems in Chengdu may have regional particularities that differ from orthopedic nursing practices in other regions of China or internationally. Fourth, only individual-level variables were included, while situational factors such as departmental staffing and leadership style were not involved.

Future research can be carried out in the following aspects: First, adopt prospective cohort studies or randomized controlled trials to clarify the causal relationship between job burnout and sleep quality, and verify the long-term effects of interventions such as mindfulness-based stress reduction on improving sleep quality and alleviating burnout among orthopedic nurses. Second, expand the geographical scope of the research sample to include orthopedic nurses in hospitals of different levels, so as to improve the extrapolation of the research results. Third, include organizational-level variables such as departmental staffing and leadership style, and construct a multi-level model to analyze their impact on nurses' burnout and sleep. Fourth, quantify workload indicators such as night shift frequency and nurse-patient ratio, and further explore their moderating role in the relationship between burnout and sleep quality.

## Conclusion

Job burnout significantly affects sleep disorders among orthopedic nurses, with emotional exhaustion having the greatest impact; those who are married, have long tenure, and have low income are at higher risk. Reducing burnout and strengthening support and incentives are key to improving sleep quality among orthopedic nurses.

## Data Availability

The raw data supporting the conclusions of this article will be made available by the authors, without undue reservation.
